# Beyond T-Cells: Functional Characterization of CTLA-4 Expression in Immune and Non-Immune Cell Types

**DOI:** 10.3389/fimmu.2020.608024

**Published:** 2020-12-15

**Authors:** Damilola Oyewole-Said, Vanaja Konduri, Jonathan Vazquez-Perez, Scott A. Weldon, Jonathan M. Levitt, William K. Decker

**Affiliations:** ^1^ Department of Pathology & Immunology, Baylor College of Medicine, Houston, TX, United States; ^2^ Michael E. DeBakey Department of Surgery, Baylor College of Medicine, Houston, TX, United States; ^3^ Dan L. Duncan Cancer Center, Baylor College of Medicine, Houston, TX, United States; ^4^ Scott Department of Urology, Baylor College of Medicine, Houston, TX, United States; ^5^ Center for Cell and Gene Therapy, Baylor College of Medicine, Houston, TX, United States

**Keywords:** CTLA-4, peripheral tolerance, immune regulation, tumor immunity, CD28

## Abstract

The immune response consists of a finely-tuned program, the activation of which must be coupled with inhibitory mechanisms whenever initiated. This ensures tight control of beneficial anti-pathogen and anti-tumor responses while preserving tissue integrity, promoting tissue repair, and safeguarding against autoimmunity. A cogent example of this binary response is in the mobilization of co-stimulatory and co-inhibitory signaling in regulating the strength and type of a T-cell response. Of particular importance is the costimulatory molecule CD28 which is countered by CTLA-4. While the role of CD28 in the immune response has been thoroughly elucidated, many aspects of CTLA-4 biology remain controversial. The expression of CD28 is largely constrained to constitutive expression in T-cells and as such, teasing out its function has been somewhat simplified by a limited and specific expression profile. The expression of CTLA-4, on the other hand, while reported predominantly in T-cells, has also been described on a diverse repertoire of cells within both lymphoid and myeloid lineages as well as on the surface of tumors. Nonetheless, the function of CTLA-4 has been mostly described within the context of T-cell biology. The focus on T-cell biology may be a direct result of the high degree of amino acid sequence homology and the co-expression pattern of CD28 and CTLA-4, which initially led to the discovery of CTLA-4 as a counter receptor to CD28 (for which a T-cell-activating role had already been described). Furthermore, observations of the outsized role of CTLA-4 in T_reg_-mediated immune suppression and the striking phenotype of T-cell hyperproliferation and resultant disease in CTLA-4^−/−^ mice contribute to an appropriate T-cell-centric focus in the study of CTLA-4. Complete elucidation of CTLA-4 biology, however, may require a more nuanced understanding of its role in a context other than that of T-cells. This makes particular sense in light of the remarkable, yet limited utility of anti-CTLA-4 antibodies in the treatment of cancers and of CTLA-4-Ig in autoimmune disorders like rheumatoid arthritis. By fully deducing the biology of CTLA-4-regulated immune homeostasis, bottlenecks that hinder the widespread applicability of CTLA-4-based immunotherapies can be resolved.

## Introduction

CD28 and CTLA-4 are homologous glycoproteins of the immunoglobulin superfamily (1). Despite their shared ability to bind CD80/B7.1 and CD86/B7.2 (B7 proteins) (2, 3), these molecules mediate opposing effects on T-cell function. While CD28 promotes T-cell activation and proliferation (4, 5), CTLA-4 is reported to dampen T-cell responses through a variety of mechanisms (6–12). Prior to activation, conventional T-cells (T_conv_) express low levels of CTLA-4, predominantly in intracellular compartments. Upon activation, CTLA-4 expression is upregulated and becomes increasingly detectable on the cell surface, peaking around 36 h post-activation (13–15). In Tregs on the other hand, transmembrane CTLA-4 is constitutively expressed and plays an integral role in Treg homeostasis and function (16–18). In general, T-cell CTLA-4 is largely constrained to intracellular expression although some surface expression may be detectable owing to the rapid, continuous shuttling of CTLA-4 between intracellular compartments and the plasma membrane (10, 19–22).

Intracellular trafficking of CTLA-4 requires the recruitment of a specific repertoire of proteins by vesicle-bound-intracellular CTLA-4 and by CTLA-4 at the plasma membrane (9, 23). These protein–protein interactions are largely mediated by the recruitment of the clathrin adaptor complexes AP-1 (at the trans-golgi) and AP-2 (at the plasma membrane) by the intracellular YVKM motif of CTLA-4 (10, 19, 20, 24, 25). Tight control of turnover is another important regulator of CTLA-4 expression and function. Specifically, lipopolysaccharide-responsive and beige-like anchor protein (LRBA) protects CTLA-4 from lysosomal degradation thus promoting its accumulation in the cytoplasm and subsequent surface expression (26). Although LRBA deficiency presents with a variable phenotype (26, 27), autoimmunity and hypogammaglobulinemia characterized by decreased Treg, total B-cell, class-switched B-cell and plasmablast frequency along with an increased proportion of circulating T_fh_ are prevalent (27–30). In these patients, CTLA-4-mediated functions can be substantially impaired (26–31).

The most widely reported mechanism of CTLA-4-mediated inhibition is its competitive binding of B7 proteins to which it has a 10–100-fold greater affinity than CD28 (32, 33). As a result, T-cells are deprived of CD28-mediated activating signals. Subsequent to binding B7 proteins, CTLA-4 has also been described to further deprive T-cells of stimulatory signaling *via* transendocytosis of B7 molecules from the surface of APCs (11, 22), inhibition of proximal T-cell receptor (TCR) signaling (6), and disruption of the c-SMAC (central supramolecular cluster) within the immunological synapse (34). The disruption of CD28 signaling is generally accepted as the major pathway through which CTLA-4 functions as evidenced by observations that fatal lymphoproliferation observed in CTLA-4^−/−^ mice is rescued by the genetic deletion of CD28 (35).

Aside from these cell-extrinsic mechanisms, CTLA-4 has also been shown to function through cell-intrinsic mechanisms. Upon binding of B7 molecules, CTLA-4 has been reported to signal through PI3K and PKC*δ* in activated conventional T-cells (23, 36, 37). In these cells, CTLA-4 signaling reinforces its previously described inhibitory role by recruiting the phosphatases SHP-2 and PP2A to the immunological synapse, thereby reversing the phosphorylation of secondary messengers by TCR co-receptors and co-stimulatory molecules (38–41). This cell-intrinsic program also serves to limit the contact-dependent suppressive capacity of Tregs through PKC-*η* signaling (7) and prevent activation-induced cell death, particularly in T_H_2 cells, by promoting Bcl2 expression while downregulating FasL (42). In addition, CTLA-4 ligation enforces a reversal of the TCR stop signal which may decrease the contact time between T-cells and APCs and could also explain the apparent ability of CTLA-4 to drive T_H_ cell migration to secondary lymphoid organs (37, 43).

## B-Cells

While the T-cell phenotype in CTLA-4^−/−^ mice is striking and largely accounts for the observed tissue destruction, B-cells in these mice also displayed a hyperactivated phenotype. This distinctive B-cell signature includes hypergammaglobulinemia as well as upregulated expression of CD86, FAS and CD5 on B-cells (44). Accordingly, CTLA-4 expression in B-cells was subsequently demonstated (31, 45–48). Coupled with reports of auto-antibody production and deficiencies in antigen-specific antibody generation in LRBA-deficient patients (29), these observations provided the rationale for studying CTLA-4 in the context of B-cell activation. Initial reports indicated that the role of CTLA-4 in limiting B-cell responses was mediated via T_fh_, T_freg_, and T_reg_ expression of CTLA-4 (49). The authors reported that CTLA-4-mediated control of B-cell activation could occur within or outside of the germinal center and could occur independently of CD80 and CD86. The experiments outlined in this report demonstrated, for the first time, that CTLA-4 could extrinsically subvert B-cell activation. The observations, however, did not preclude cell-intrinsic mechanisms of CTLA-4-mediated constraint of B-cell responses.

Aside from the expression of CTLA-4 on human B-cell chronic lymphocytic leukemia (discussed in ‘Tumors’ section), the expression of CTLA-4 on B1 and B2 B-cells has been sparsely studied but well-validated in mice and humans. The stimuli which drive B2 B-cell expression of CTLA-4 remain controversial, however. Some reports assert that B2 B-cell CTLA-4 expression may be driven by PMA, LPS + IL-4, or CD40 + IL-4 stimulation (45–47), while others determined that B2 B-cell CTLA-4 expression was strictly thymus-dependent (48, 50) and could not be driven by Protein Kinase C activation, cytokine stimulation, or bacterial products alone. Further studies have so far failed to fully resolve the impact of these different stimuli on B-cell CTLA-4 expression. Within the B-cell compartment, it is important to determine the physiology of CTLA-4 expression in both B1 and B2 B-cells, each of which undertakes distinct roles within the broader immune response. In particular, B1 B-cells participate in innate immune responses by constitutively generating low affinity, highly cross-reactive antibodies (referred to as natural antibodies) in a T-cell-independent manner. These cells are self-renewing and primarily localized to pleural and peritoneal tissues. Consequently, B1 B-cells and their antibodies are the brokers of innate humoral responses. B2 B-cells, on the other hand, are commonly taken to be the conventional B-cell subset. They participate in the adaptive immune response, generating highly specific antibodies in a T-cell-dependent or independent manner during the later phase of an immune response. In contrast to B1 B-cells, these cells are generally located in the bone marrow and secondary lymphoid organs. Additionally, B2 B-cells may be recruited to peripheral organs during an immune response. In B2 B-cells, surface CTLA-4 expression was reported to be fivefold lower than observed in activated T-cells and was transient, peaking within 48 h and disappearing by 96 h after stimulation (48). Importantly, the transfer of CTLA-4 from T-cells to co-cultured B2 B-cells was ruled out by virtue of the observation that CTLA-4^−/−^ B2-cells remain negative upon co-culture with CTLA-4-sufficient T-cells. Although B2 B-cell CTLA-4 was not required for B-cell differentiation or homeostasis in mice (48), it was reported to restrain isotype switching. Particularly, CTLA-4 ligation limited the production of antigen-specific and total IgM, IgG and IgE (46, 48) but not of natural antibodies (48). CTLA-4 ligation by human B2 B-cells led to the downregulation of surface and secreted IgG as well as IL-8, TNFα and IL-10 (45). In contrast, the role of CTLA-4 in B1 B-cell development and function remains undefined, although its expression has been reported (51). Given the reported lack of alterations to natural antibody production in CTLA-4^−/−^ mice, it is possible that B1 CTLA-4 exerts a cell-extrinsic inhibitory function on the immune response.

Whether B-cell CTLA-4, in fact, possesses cell-extrinsic functions remains to be determined. As previously stated, LRBA-deficient patients and mice (with limited surface CTLA-4 expression) present with diminished total B-cell, IgG class-switched B-cell and plasmablast numbers, accompanied by increased frequency of circulating T_fh_ and the generation of autoantibodies, despite reported hypogammaglobulinemia (26, 27, 29, 52). While the administration of CTLA-4-Ig alleviates the overall clinical phenotype and reduces T_fh_ dysfunction in LRBA-deficient patients (26), it is unclear whether the observed therapeutic effects may be solely attributed to restored Tfh control of B-cell activation or by the impact of CTLA-4-Ig directly on B-cells. As such, it remains to be determined whether B-cell CTLA-4 possesses cell-intrinsic functions. Given the co-expression of CTLA-4 and B7 on B-cells, it may be valuable to clarify all pertinent functions of these molecules in the context of B-cell biology since they pertain to both B-cell:B-cell interactions as well as B-cell:T-cell interactions within secondary lymphoid organs. It also remains unclear whether specific B-cell subsets such as marginal zone B-cells and memory B-cells possess distinct CTLA-4-mediated functions during an ongoing primary or recall immune response. These questions might be resolved through further studies utilizing B-cell-specific deletions of CTLA-4 coupled with CTLA-4-Ig models of rescue as well as studies of bone marrow chimeric mice. Additionally, the specific mechanisms which drive B-cell CTLA-4 expression and function are yet to be fully understood beyond their T-cell dependence. Specifically, the B-cell:T-cell interactions and resultant signaling cascades that drive CTLA-4 expression in B-cells are unknown. Furthermore, the mechanisms through which B-cell CTLA-4 restrains antigen-specific IgM and IgE production have not been determined, although STAT6 and NF*κ*B activation were shown to be suppressed upon B-cell CTLA-4 ligation. Furthermore, the expression and function of CTLA-4 on non-malignant human B-cells has not been investigated. These mechanisms could prove important for elucidating pathways involved in the generation of allergic and autoinflammatory diseases, particularly those with confirmed B-cell involvement.

## NK Cells

Accounts of CTLA-4 expression in NK cells are even more scarce than those in B-cells. Presently, our knowledge is based primarily on one mouse and one human study. Interestingly, although both studies are in agreement regarding the conditions which drive the expression of CTLA-4 in their respective model systems, they come to disparate conclusions regarding its function in NK cells. In mouse studies conducted by the Cerwenka group (53), only IL-2-primed NK cells and tumor-infiltrating NK cells were observed to express CTLA-4. Similar to T-cells, CTLA-4 expression in NK cells was largely constrained to intracellular compartments. Moreover, CTLA-4 levels in IL-2-primed mouse NK cells could be modulated by cytokine treatment in their *in vitro* culture system. In particular, the combination of IL-12 and IL-18 but not IL-15 synergized with IL-2 to further enhance CTLA-4 expression while TGF-*β* served to counteract CTLA-4 expression. These observations were recapitulated in human studies carried out by Lougaris et al (54). Similarly, IL-2-primed human NK cells expressed intracellular CTLA-4, which could be further increased by IL-12 + IL-18 treatment. Presumably, the impact of IL-12 and IL-18 on the expression of NK CTLA-4 is of physiological relevance, since these cytokines have already been described as important modulators of NK cell phenotype, especially in the context of NK-DC crosstalk. Specifically, DCs (and macrophages) secrete IL-12 and IL-18 which synergize to boost NK cell-mediated cytotoxicity, IFN-*γ* production, and IL2R*α* expression (55, 56). Regarding the function of NK CTLA-4, there is a disagreement between the mouse and human data. CTLA-4 ligation was shown to be inhibitory to mouse NK cell function (53) while, conversely, the absence of such ligation in human NK cells resulted in limited function (54). Specifically, in mouse NK cells, CTLA-4 was shown to limit the expression of IFN-*γ* upon B7 ligation or co-culture with mature dendritic cells.

When mature DCs were pre-treated with CTLA-4-Ig before co-culture with NK cells, IFN-*γ* expression by NK cells was partly inhibited. This experiment indicated that CTLA-4 may mediate its inhibitory role partly through B7 blockade in mice (53). However, the role of CTLA-4 in NK cell cytotoxicity was not examined in this study. Although CTLA-4-regulated IFN-*γ* likely plays a salient role in NK-mediated immunity, understanding the role of CTLA-4 in NK-mediated cytotoxicity could be an equally important phenomenon that remains unexamined.

In human NK cells, CTLA-4 function was examined in the contexts of NK cell development, IFN-*γ* production, and cytotoxicity (54). Here, CTLA-4 haploinsufficiency was not observed to affect NK cell development; rather, it was shown to limit IFN-γ expression in response to cytokine stimulation as well as degranulation in response to target K562 cells. This was surprising given the inhibitory role already described for mouse NK CTLA-4 and both mouse and human T-cell CTLA-4. In light of the disagreement between human and mouse NK cell studies as well as the variable expression of CD28 (57, 58) on human NK cells [unlike mouse NK cells (53)], the role of NK cell CTLA-4 in the immune response of each organism must be thoroughly validated. It will be important to determine the function of NK CTLA-4 particularly at the early phase of an immune response, the time at which NK cells are most often activated.

## Monocytes and Dendritic Cells

The interaction between dendritic cells (DCs) and T-cells is critical to determining the type, strength, and likely the duration of T-cell-dependent immune responses. Not only do dendritic cells prime *de novo* T-cell activation, they also contribute to the dampening of T-cell responses through negative selection in the thymus and can induce tolerance or anergy in the periphery (59–61). While the expression of CTLA-4 has been reported for T-cells and DCs, investigations into the origins and function of T-cell CTLA-4 are far more numerous than similar explorations of DC CTLA-4. In addition to the regulation of T-cell responses, the interaction between DCs and NK cells as well as non-immune cells is important to regulating the overall immune response. While it has been demonstrated that ligation of CTLA-4 molecules on the surface of DCs can engender a tolerogenic DC phenotype involving IL-10 and IDO expression (62, 63), the direct impact of DC CTLA-4 on immune cell phenotype is unknown. Initial reports demonstrated that freshly isolated CD14^+^ human monocytes could express CTLA-4 (64). Like Tcell CTLA-4, this was largely restricted to intracellular compartments and could be upregulated by activation of Protein Kinase C (PKC) *via* treatment of monocytes with PMA and IFN-*γ* (64). These early observations made no conclusions as to whether these activated monocytes were differentiated into dendritic cells or macrophages, both of which could be derived from cultured monocytes. More recent investigations, however, determined that bone marrow monocyte-derived dendritic cells were capable of upregulating soluble and transmembrane isoforms of CTLA-4 upon maturation with LPS, Poly I:C or a cocktail of inflammatory cytokines (63, 65). Notably, Immature DCs expressed little to no intracellular or surface CTLA-4. The expression of CTLA-4 in DCs was also determined to delineate a novel subset of regulatory DCs present in hepatocellular carcinoma patients. In these patients, CD14^+^ CTLA-4^+^ DCs also expressed the inhibitory molecules PD-1, IDO and IL-10 (62). Cross-linking of CTLA-4 in these DCs further enhanced the expression of IDO and IL-10, which presumably contributed to immunosuppression and subsequent tumor escape.

**Figure 1:**
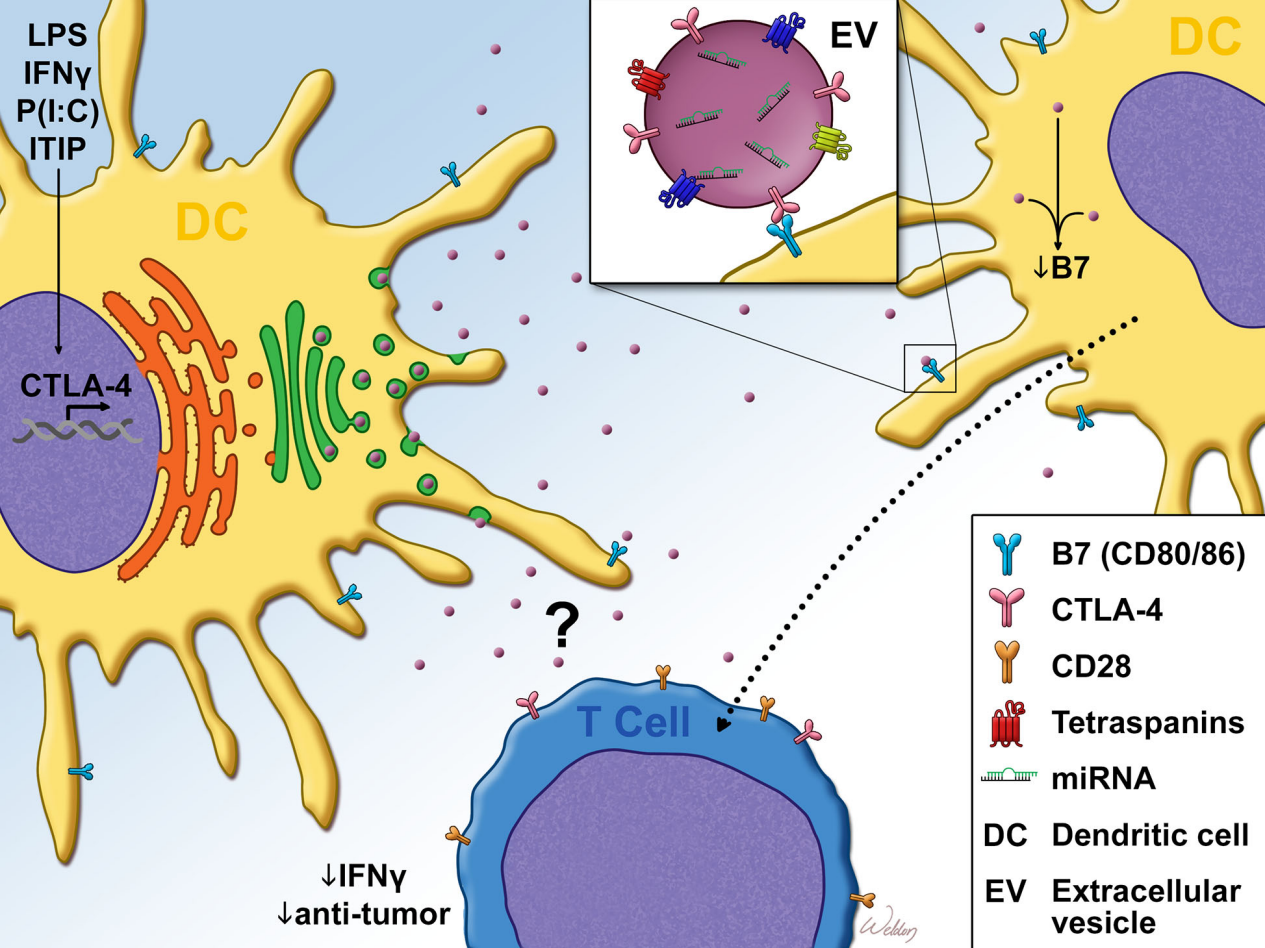
Dendritic cell CTLA-4 suppresses T-cell responses Cytokine or TLR agonist stimulation promotes dendritic cell (DC). maturation as well as the expression of CTLA-4. Unlike T-cell CTLA-4 which is predominantly localized to the plasma membrane, DC CTLA-4 expression is diffuse, with some localization to the trans-golgi. After leaving the golgi, DC CTLA-4 is packaged into CD63^+^ Rab5^+^ extracellular vesicles (EVs), which are then exported from the cell. Once in the extracellular space, bystander DC take up the EVs in a B7 (CD80/CD86)-dependent manner. Uptake of EVs in this fashion resulted in the downregulation of surface B7. DC CTLA-4 has also been reported to limit IFN-*γ* expression as well as anti-tumor efficacy of CD8^+^ T-cells. This T-cell phenotype may be regulated through the direct impact of the CTLA4^+^ EVs on T-cells or through bystander DC that take up CTLA4^+^ EVs, as evidenced by DC-T cell co-culture experiments and *in vivo* DC vaccine studies. LPS, Lipopolysaccharide; IFN-*γ*, Interferon-gamma; P(I:C), Polyinosinic:polycytidylic acid; ITIP, pro-inflammatory cytokine cocktail which consists of: IL-1β, TNFα, IL-6 and Prostaglandin E_2_.

Due to the lack of homogeneity within the DC compartment, it became necessary to define which DC subsets expressed CTLA-4. It was determined that while both CD1a^+^ and CD1a^−^ dendritic cells expressed intracellular CTLA-4 upon maturation, only CD1a^+^ DCs expressed measurable levels of surface CTLA-4 (66). Further functional characterization of DC CTLA-4 followed, leading to the determination that ligation of the molecule inhibited AP-1 and NFkB activity and suppressed DC maturation and proliferation (64). Such ligation also promoted the expression of IL-10 and suppressed IL-8 release and T-cell responses in vitro. Accordingly, CTLA-4 ligation did not significantly impact antigen presentation, since the molecule was minimally expressed on immature dendritic cells (64). Further studies by Halpert et al. demonstrated for the first time that CTLA-4 expressed by both human and mouse bone marrow or monocyte-derived DC was capable of exerting cell-extrinsic effects on neighboring dendritic cells. This work determined that CTLA-4 was packaged into extracellular vesicles (EVs) which could be taken up by neighboring dendritic cells. Upon uptake of these CTLA-4^+^ EVs, B7 expression on the surface of recipient dendritic cells was significantly downregulated (**Figure 1**). Furthermore, the expression of CTLA-4 by a dendritic cell vaccine was found to impact antitumor efficacy (65). siRNA-mediated knockdown of CTLA-4 in a DC vaccine significantly improved tumor control and survival, correlating with vastly increased frequency of activated CD8^+^ T-cells. This result was verified in human monocyte-derived DC which, following treatment with CTLA-4 siRNA, stimulated the proliferation of far greater numbers of IFN-*γ* ^+^ CD8^+^ T-cells and far fewer CD4^+^ CD25^+^ Foxp3^+^ phenotypic T_regs_. Accordingly, the same authors subsequently demonstrated that secretion of microvesicular CTLA-4 by dendritic cells plays a critical role in the regulation of T_H_ polarization and promulgation of T_H_1 immunity (67). Although some features of DC CTLA-4 physiology have been determined, several important aspects are yet to be resolved. For instance, the role of CTLA-4 on the development of DCs and on DC-mediated negative selection of T-cells is unknown. Likewise, the underlying mechanisms which drive the effects of DC CTLA-4 and CTLA-4^+^ EVs on other dendritic cells, T-cells and anti-tumor immunity are unknown. While the expression of DC CTLA-4 in bone marrow-derived DCs has been thoroughly validated, it is unclear which *in vivo* DC subsets express CTLA-4 in a similar fashion. Our unpublished observations from mouse splenic DCs have revealed that the expression of CTLA-4 by DC subsets is indeed differential. We observed that cDC1 cells (conventional DC type 1) expressed significantly greater intracellular CTLA-4 levels than cDC2 cells, monocytes, monocyte-derived DCs and plasmacytoid DCs (pDCs). While monocytes expressed undetectable levels of intracellular CTLA-4, the remaining subsets expressed comparable levels of CTLA-4. This would mark the first report of CTLA-4 expression in cDC1, cDC2 and pDC subsets. Our report, in contrast to others (62–66), differs with regard to the distinct absence of CTLA-4 expression in monocytes. This disparity likely stems from our stringent gating of mouse DC subsets [as previously described (68)], which differs from the CD14-predicated gating of monocytes in previous reports. These observations, coupled with reports of CTLA-4 expression in MDSCs and M2 macrophages in HNSCC tumors (69), underscore the importance of investigating the physiology of CTLA-4 within the monocyte lineage, particularly its utility in type I and type II T-cell responses as well as in T-cell-independent responses.

## Tumors

The earliest report of CTLA-4 expression in tumors came from observations made in leukemias as well as Hodgkin’s and non-Hodgkin’s lymphomas (NHL) (70). CTLA-4 was detected on acute and chronic myeloid leukemias, large granular lymphocytic leukemia and malignant follicular B-cells of patients with low-grade B-cell lymphoma as well as non-malignant reactive B-cells in NHL patients. While CTLA-4 was detected in both B-cell chronic and acute lymphocytic leukemia, it was reported only in T-cell acute and not chronic lymphocytic leukemia (47, 70). CTLA-4 expression has also been reported in the lymphoblastic Raji and Daudi cell lines (71).

In patients with Hodgkin’s disease, CTLA-4 was not detected on any neoplastic lymphocytes and was notably absent in Reed-Sternberg cells (70). Although CTLA-4 is known as a negative regulator of T-cell proliferation, CTLA-4 expression was paradoxically detected on the malignant T-cells of patients with peripheral but not lymphoblastic or anaplastic Non-Hodgkin lymphomas (NHL) (70). While this expression of CTLA-4 on neoplastic peripheral T-cells would be in keeping with the inducible expression of CTLA-4 on activated T-cells, it calls into question the mechanisms that underlie the seeming loss of intrinsic CTLA-4-mediated control of T-cell proliferation (72, 73) as well as the activating signals which drive CTLA-4 expression in neoplasms. Perhaps CTLA-4 expression promotes tumor escape in the periphery (by inhibiting anti-tumor responses) but is incapable of inhibiting malignant lymphocyte proliferation through well-documented cell-intrinsic modes of immune suppression. Although these questions are yet to be resolved, observations derived from studies of B-cell CTLA-4 in CLL have revealed important clues that could mirror the physiology of CTLA-4 in T-cell malignancies (74, 75). It was observed that B-cell CTLA-4 was most highly expressed in the bone marrow and peripheral blood in comparison to lymph node B-cells where expression was significantly lower (76). In these malignant B-cells, CTLA-4 expression was constitutive and intracellular but could be driven to the surface *via* co-culture with activated T-cells. Here, neoplastic B-cell CTLA-4 successfully mediated transendocytosis of surface CD80 (from CD80-GFP^+^ cells) and downregulated IL-2 production by co-cultured T-cells (75). Functional characterization of tumor CTLA-4 function proceeded with the study of cell-intrinsic effects of CTLA-4 on B-cells in CLL. Here, decreased expression of CTLA-4 correlated with increased expression of c-Myc, phospho-STAT1, NFATc2, phospho-c-Fos, and Bcl2, molecules which presumably are downstream of B-cell Receptor (BCR) activation (76). Accordingly, CTLA-4 downregulation also correlated with decreased apoptosis in CLL B-cells. These lines of evidence suggest that neoplastic B-cell CTLA-4 is functionally suppressive through both cell-intrinsic and cell-extrinsic pathways. It would seem that malignant B-cell CTLA-4, at least, is capable of dampening T-cell-mediated anti-tumor immune responses while paradoxically limiting B-cell proliferation and survival. Perhaps there remains sufficient heterogeneity, with regard to CTLA-4 expression, such that lymphoproliferation can occur with CTLA4^−^ malignant B-cells in the lymph node with concurrent inhibition of anti-tumor responses by CTLA-4^+^ malignant B-cells in peripheral blood or bone marrow, thus favoring overall tumor progression.

The prognostic value of B-cell CTLA-4 in CLL is not yet clear-cut. Some reports suggest that CTLA-4 expression promotes the survival of leukemic B-cells and correlates with disease progression (74, 76), while others assert that CTLA-4 expression may be a marker of improved outcomes (77). This prognostic mirrors the disparate conclusions that have been drawn regarding the expression of CTLA-4 in non-immune cell malignancies. In some breast cancers (78, 79), thyomomas (80), esophageal carcinomas (81) and nasopharyngeal carcinomas (82), tumor expression of CTLA-4 correlated with poor prognosis while no relationship to survival was observed with testicular germ cell tumors (83). Although the basis for the negative correlation of CTLA-4 expression to prognosis was not determined in the relevant studies, it is possible (perhaps even probable) that such poor prognoses were mediated by the direct suppression of immune effector cell function by CTLA4^+^ tumor tissue. A cogent demonstration of this immune suppression was demonstrated in breast cancer cells, which have been shown to express CTLA-4. In this study, CTLA-4^+^ breast cancer cells suppressed dendritic cell maturation, antigen presentation, and inflammatory cytokine expression, ultimately dampening T_H_1 and CTL responses (79). Yet, in non-small cell lung cancer (NSCLC) (84) and when expressed in the hepatic hilar region of extrahepatic bile duct cancer patients (85), studies determined that CTLA-4 correlated with improved survival. However, later studies showed that disease-specific survival did not correlate with primary tumor CTLA-4 expression (86). Rather, the prognostic value of CTLA-4 in NSCLC could only be revealed when patients were stratified by disease subtype. Here, metastatic lymph node expression of CTLA-4 correlated with poor prognosis while soluble CTLA-4 expression predicted improved prognosis in squamous cell carcinoma. CTLA-4 is reported to be highly expressed in most human and mouse melanoma cell lines as well as normal melanocytes, many primary melanomas, and melanoma stem cells with conflicting data suggesting both immune and non-immune-related roles in tumorigenesis (87–89). Altogether, the disparate effects of tumor CTLA-4 expression make a strong case for studying the effects of CTLA-4 expression within each separate microenvironment in each cancer type and at different stages of cancer progression, rather than extrapolating results from one cancer type.

## Non-Hematopoietic/Non-Cancer

The study of CTLA-4 expression and function has so far been centered on hematopoietic lineages and anti-tumor responses. While the immunological role of CTLA-4 is indisputable, it is clear that the function of CTLA-4, particularly in the maintenance of tissue homeostasis and tolerization at immune-privileged sites, may be more nuanced than has been described. Moreover, immunological roles such as the ability to secrete cytokines and antimicrobial peptides in response to immune stimuli have been observed in non-hematopoietic cells (90–94). Non-hematopoietic cells have also been reported to engage hematopoietic cells, direct their differentiation, and drive autoimmune disease development (91, 93–95). Importantly, CTLA-4 polymorphisms have been linked to autoimmune conditions including type I diabetes (96, 97), Hashimoto's thyroiditis (97, 98), systemic lupus erythematous (99, 100) and celiac disease (101), indicating possible roles for non-hematopoietic tissue CTLA-4 in the ontogeny and progression of autoimmune disease. As such, investigations into the expression pattern and function of CTLA-4 in non-tumor and non-hematopoietic tissue is warranted. In this regard, CTLA-4 expression has been reported in mesenchymal stem cells and placental fibroblasts (102, 103) as well as the aforementioned normal melanocytes. Additionally, stimulation of cultured muscle cells with IL-1α IFN-*γ* or IL-2 + anti-CD28 promoted CTLA-4 expression (104).

In general, human mesenchymal stem cells (MSCs) are highly immunosuppressive and have been theorized to exert their effects through a variety of mechanisms (reviewed elsewhere) (105). While the release of HLA-G and factors such as nitric oxide and PGE_2_ have been identified as mechanisms of MSC-induced immunosuppression, no consensus has been reached regarding the dominant mode of immune inhibition. These cells predominantly expressed the full-length CTLA-4 (flCTLA-4) and secreted soluble CTLA-4 (sCTLA-4), although other isoforms were also detected. MSC CTLA-4 was determined to inhibit TNFα production by PHA-activated PBMCs, a phenomenon that was boosted by hypoxic conditions. Accordingly, hypoxic culture conditions significantly upregulated the expression of flCTLA-4 and sCTLA-4 in MSCs (103).

Efforts to define the immunosuppressive landscape of the maternal-fetal landscape during pregnancy led to the discovery that fetal cells expressed CTLA-4 during gestation. Notably, cultured human placental fibroblasts and mesenchyme as well as placentas from all gestational time points expressed CTLA-4 (102).

In the same vein, viral infections, such as with Human Papilloma Virus (HPV) have been demonstrated to imprint an immunosuppressive phenotype upon the host organism (106, 107). HPV, in particular, has been shown to impair host NK and T-cell antiviral responses through a variety of mechanisms including Treg recruitment, inhibition of DC maturation and suppression of NK cell cytotoxicity. In keeping with this theme, the HPV protein HPVE7, has been reported to induce CTLA-4 expression in human cervical epithelium and keratinocytes by promoting H3K36me2 at the CTLA-4 promoter (108). While no functional characterization of CTLA-4 in this context has been carried out, the authors hypothesize that it may contribute to the immunosuppressive phenotype and subsequent immune escape engendered by HPV.

## Conclusions

The original characterizations of CTLA-4 function and biology were driven by the dramatic T-cell-intrinsic phenotype observed in global knockout mice, animals that were born normal but died in the early postnatal period of massive and uncontrolled lymphoproliferation (44, 109). When follow-up studies that included concomitant global deletion of CD28 (110) as well as re-expression of CTLA-4 in null mice from the T-cell-specific Lck promotor (111) both rescued this dramatic but early phenotype, there remained little impetus to look for additional mechanisms of action outside the T-cell compartment. In contrast to this early paradigm, studies over the last two decades have demonstrated both constitutive and inducible expression of CTLA-4 in a broad distribution of tissues and cell types (**Table 1**). This expansive pattern of expression and the circumstances under which induction may occur have necessitated a reevaluation of the role that CLTA-4 plays in global immune regulation. A variety of convincing studies (**Table 1**), particularly those in dendritic cells and tumors, now demonstrate that CTLA-4 plays nonredundant and critical roles in thymic development, T-cell priming, peripheral tolerance, and a variety of other critical immunoregulatory functions. Further, it appears likely that additional roles for this central and highly versatile molecule will be uncovered as additional studies progress.

**Table 1:** Summary of Evidence of non-T-cell CTLA-4 by Cell Type.

Cell type		Experimental Evidence	Controls	Anti-CTLA-4 clone	Reference
B-cells	Resting and activated human and mouse B-cells	A. Flow cytometry (45–48, 50) B. Immunoprecipitation + western blot (50) C. RT-PCR (48) D. Analysis of bone marrow- chimeric mice (48) E. Functional analysis of anti-CTLA-4-mediated cross-linking (46)	CTLA-4–Ig-transfected J558L murine myeloma (47), T-cells and Isotype controls (45–48, 50).	BNI3 (47)	(45–48, 50)
	B1 B-cells (mouse)	*Post-hoc* analysis of publicly-available B-cell microarray datasets	Not provided	Not provided	(51)
NK Cells	IL-2-primed mouse NK cells	A. Flow cytometry B. qRT-PCR C. Functional analyses using CTLA-4-Fc and NK cells from CTLA-4 KO mice	Isotype control for functional analyses, isotype controls for flow cytometry.	UC10-4F10-11 and UC10-4B9	(53)
	IL-2-primed human NK cells	Flow cytometry	CTLA-4 haploinsufficient patient samples, isotype control.	Not provided	(54)
Monocytes and DCs	CD14^+^ Human monocytes	A. Flow Cytometry (63, 64) B. Fluorescence microscopy (64) C. Western blot (64) D. RT-PCR (64)	CTLA-4–Ig-transfected J558L murine myeloma (63), T-cells (64), isotype control (47, 63, 64), C33a cervical cancer cell line and surface staining of resting T-cells (64).	BNI3 (63); N‐19 and AF‐386‐PB (64)	(47, 63, 64)
	Human monocyte-derived dendritic cells	A. Flow Cytometry (63, 65, 66) B. Western blot (65, 66) C. Confocal microscopy (65) D. siRNA knockdown (65) E. Immunoprecipitation (65) F. RT-PCR and PCR product sequencing (65, 66) G. Functional analyses (63, 65, 66)	PBMC (63, 65, 66), T-cells (65), isotype control (63, 65, 66), competitive antibody binding, non-targeting siRNA (65).	BNI3 and A3.6B10.G1 (65); 48815, 14D3 and agonist 3D5 (63)	(63, 65, 66)
	Mouse bone marrow-derived dendritic cells	A. siRNA knockdown and western blot B. siRNA knockdown with functional analysis	Non-targeting siRNA	Not provided	(63, 65, 66)
MDSCs and Macrophages	Mouse MDSCs (CD11b^+^Gr-1^+^) and M2 macrophages (CD11b^+^F4/80^+^)	A. Flow cytometry B. Fluorescence microscopy	Isotype control	Not provided	(69)
Granulocytes	G-CSF-treated human granulocytes	Flow cytometry	CTLA-4–Ig-transfected J558L murine myeloma, T-cells, isotype controls.	BNI3	(47)
CD34^+^ hematopoietic stem cells	GM-CSF-treated and untreated CD34^+^ stem cells	A. Flow cytometry B. Immunohistochemistry	CTLA-4–Ig-transfected J558L murine myeloma, T-cells, isotype control.	BNI3	(47)
Immune cell malignancies	Non-Hodgkin’s B-cell lymphoma (follicular)	Immunohistochemistry	CTLA-4-transfected cells and reactive lymph node samples, Jurkat cells	BNI3	(70)
	T-cell lymphoma (peripheral), T-CLL	Immunohistochemistry	CTLA-4-transfected cells and reactive lymph node samples, Jurkat cells	BNI3	(70)
	B-Chronic lymphocytic leukemia (human and TCL1 transgenic mice)	A. Microarray (75–77) B. Flow Cytometry (47, 75–77) C. RT-PCR (75, 76) D. siRNA knockdown (76) E. Western blot (76)	Isotype control (75, 76), CTLA-4–Ig-transfected J558L murine myeloma (47), T-cells (47), Jurkat cells (75), scrambled siRNA (76)	BNI3 (47, 75), UC10-4F10-11 (75), 9d9 (75),	(47, 75–77)
	AML, CML, B-ALL, T-CLL, LGL	A. Flow cytometry	CTLA-4–Ig-transfected J558L murine myeloma, T-cells, isotype controls.	BNI3	(47)
	Raji and Daudi B- lymphoblastoid cell lines	A. Flow Cytometry (47, 71) B. RT-PCR + nested PCR (47)	T-cells (47, 71), CTLA-4–transfected J558L murine myeloma (47) and isotype controls (47, 71).	BNI3 (47)	(47, 71)
Non-immune cell malignancies	Human breast cancer cells and cell lines (MDA-MB-231 MCF-7 SKBR3 and T47D)	A. Flow cytometry (79) B. Functional analyses with antibody blockade (79) C. Immunohistochemistry (78, 112)	Isotype control (79), human tonsil section (78, 79, 112), non-neoplastic breast epithelial tissue (78), primary antibody solution pre-cleared with CTLA-4 peptide (78) and sections stained with primary antibody-free diluent (78)	14D3 (79), bs-1179R (78), F8 (112)	(78, 79, 112)
	Melanoma	A. Flow cytometry B. Immunohistochemistry C. qRT-PCR D. ELISA (soluble CTLA-4)	Isotype control	14D3, Ipilimumab	(113)
	Esophageal carcinoma	Immunohistochemistry	Not provided	EPR1476	(81)
	Nasopharyngeal carcinoma	Immunohistochemistry	Not provided	Polyclonal antibody (251548 Abbiotec),	(82)
	Non-small cell lung cancer (NSCLC)	Immunohistochemistry	Normal human placental tissue (86), brain tissue (86), melanoma tissue (84) and isotype control (84, 86)	14D3 (84, 86)	(84, 86)
	Cholangiocarcinoma (extrahepatic bile duct cancer)	Immunohistochemistry	Not provided	Polyclonal (Abcam ab151773)	(85)
	Testicular germ cell cancer	A. Immunohistochemistry B. Analysis of TCGA datasets (mRNA)	Normal human placenta and tonsil tissue	Polyclonal (Thermofisher)	(83)
Non tumor/non-hematopoietic	Mesenchymal stem cells	A. RT-PCR B. Western blot C. Flow cytometry D. Soluble CTLA-4 ELISA E. Functional studies with CTLA-4 Ig blockade	Isotype control	BNI3	(103)
	Placental fibroblast	A. RT-PCR B. Immunohistochemistry	Isotype control and activated T-cells	Not provided	(102)
	Keratinocytes and cervical epithelium (HPVE7-induced)	A. Microarray B. RT-PCR C. Western blot D. Flow cytometry E. Immunohistochemistry	Isotype control	BNI3	(108)

## Author Contributions

DO-S, VK, JV-P, JL, and WD all collaborated in the writing and editing of this manuscript. SW provided the original artwork. All authors contributed to the article and approved the submitted version.

## Funding

This work was funded in part by NIH R01 AI127387 to WD.

## Conflict of Interest

Institutional policy requires VK and WD to declare their ownership stakes in Diakonos Research, Ltd.

The remaining authors declare that the research was conducted in the absence of any commercial or financial relationships that could be construed as a potential conflict of interest.
